# Pepper Mild Mottle Virus as a Potential Indicator of Fecal Contamination in Influents of Wastewater Treatment Plants in Riyadh, Saudi Arabia

**DOI:** 10.3390/microorganisms11041038

**Published:** 2023-04-15

**Authors:** Saleh Eifan, Khalid Maniah, Islam Nour, Atif Hanif, Mohamed Taha Yassin, Ibrahim Al-Ashkar, Islem Abid

**Affiliations:** 1 Botany and Microbiology Department, College of Science, King Saud University, Riyadh 11451, Saudi Arabiaahchaudhry@ksu.edu.sa (A.H.); myassin2.c@ksu.edu.sa (M.T.Y.); iabid@ksu.edu.sa (I.A.); 2 Department of Plant Production, College of Food and Agriculture Sciences, King Saud University, Riyadh 11451, Saudi Arabia; ialashkar@ksu.edu.sa

**Keywords:** pepper mild mottle virus, human adenovirus, fecal indicators, wastewater, viral surrogate, meteorological factors, concentration

## Abstract

Several indicators of fecal pollution in water resources are continuously monitored for their reliability and, of particular interest, their correlation to human enteric viruses—not justified by traditional bacterial indicators. Pepper mild mottle virus (PMMoV) has recently been proposed as a successful viral surrogate of human waterborne viruses; however, in Saudi Arabia there are no available data in terms of its prevalence and concentration in water bodies. The concentration of PMMoV in three different wastewater treatment plants (King Saud University (KSU), Manfoha (MN), and Embassy (EMB) wastewater treatment plants (WWTP)) was measured using qRT-PCR during a one-year period and compared to the human adenovirus (HAdV), which is highly persistent and considered an indicator for viral-mediated fecal contamination. PMMoV was found in ~94% of the entire wastewater samples (91.6–100%), with concentrations ranging from 62 to 3.5 × 10^7^ genome copies/l (GC/l). However, HAdV was detected in 75% of raw water samples (~67–83%). The HAdV concentration ranged between 1.29 × 10^3^ GC/L and 1.26 × 10^7^ GC/L. Higher positive correlation between PMMoV and HAdV concentrations was detected at MN-WWTP (*r* = 0.6148) than at EMB-WWTP (*r* = 0.207). Despite the lack of PMMoV and HAdV seasonality, a higher positive correlation (*r* = 0.918) of PMMoV to HAdV was recorded at KSU-WWTP in comparison to EMB-WWTP (*r* = 0.6401) around the different seasons. Furthermore, meteorological factors showed no significant influence on PMMoV concentrations (*p* > 0.05), thus supporting the use of PMMoV as a possible fecal indicator of wastewater contamination and associated public health issues, particularly at MN-WWTP. However, a continuous monitoring of the PMMoV distribution pattern and concentration in other aquatic environments, as well as its correlation to other significant human enteric viruses, is essential for ensuring its reliability and reproducibility as a fecal pollution indicator.

## 1. Introduction

The contamination of water resources by fecal matter or wastewater treatment plant discharge is continuously posing a global health burden [1]. Wastewater is an appropriate environment for the incidence of pathogenic microbes, including viruses, protozoa, enteric bacteria, parasitic worms, and their eggs [2]. The organic matter in wastewater was reduced through wastewater treatment plants (WWTPs), which acts as protective barriers between the environment and communities [3]. Moreover, fecal wastes are considered the main elements of domestic sewage and, consequently, a major source of human pathogens in wastewater [4]. In this context, enteric pathogens pose a potential risk to human health, including abdominal pain, diarrhea, nausea, cramping, and vomiting [5]. Coliform bacteria are commonly utilized as indicators for fecal pollution; however, prior studies have shown that the presence of enteric viruses after treatment was not reflected by coliform bacteria [6]. Furthermore, various drawbacks regarding the usage of coliform bacteria as indicators have been reported, including low specificity and reliability, as well as vulnerability to varied treatment processes and ambient conditions [7]. On the contrary, somatic coliphages, F-specific coliphages, and bacteriophages were potentially suggested as prominent indicators of fecal pollution because of their high concentrations in wastewater, similar survival rates to enteric viruses, and differential prevalence in human and animal gut microbiota [8]. However, there are significant drawbacks to utilizing bacteriophages as water quality indicators, including phage-mediated horizontal gene transfer, phage concentration decline, and bacterial resistance evolution [9]. 

Another probable fecal pollution indicator is pepper mild mottle virus (PMMoV), a plant virus belonging to the Tobamovirus genus in the Virgoviridae family, recently reported to be the most predominant RNA virus in human feces [10]. PMMoV is classified as a rod-shaped, non-enveloped virus with 312 nm size and encapsidating a 6.4-kb positive-sense single-stranded RNA genome that was first sequenced in 1991 [11]. PMMoV levels in feces are high, ranging from 10^5^ to 10^10^ copies per gram of feces (dry weight) [12]. Although PMMoV was occasionally found in feces from cows, geese, seagulls, and chickens, the viral concentrations in fecal samples of animal origin were significantly lower (3–4 logs) than in human feces [13]. In addition, PMMoV particles can survive different stages of food processing, which involves exposure to high-temperature degrees and low water activity [14]. PMMoV was discovered in environmental water samples for the first time in 2009 [15]. According to a recent study conducted in Costa Rica, the specificity of the PMMoV qPCR signal for domestic fecal wastewater was 100%, compared to 94% for that of HF183, which is a human-specific marker for Bacteroides [16]. The main advantage of using PMMoV as a biomarker for the accurate detection of fecal pollution in wastewater is its higher stability compared to other human enteric viruses in aquatic environments [17]. On the other hand, enteric viruses, such as those belonging to the Adenoviridae family, are known to be common in raw sewage and are transmitted via the fecal–oral pathway [18]. For example, human adenoviruses were reported previously to be found in high concentrations in domestic sewage water [19]. Furthermore, human adenoviruses (HAdVs) were found to have the ability to survive during different treatment processes [20]. The different serotypes of adenovirus were highly resistant to UV when compared to the other waterborne viruses, including rotaviruses, caliciviruses, echoviruses, and coxsackieviruses [21]. Collectively, adenoviruses were frequently used as indicators of fecal pollution of wastewater owing to their environmental stability [22]. However, human adenovirus is considered a principal causative agent of a wide spectrum of gastrointestinal infections in humans. Therefore, PMMoV is more advantageous than adenovirus because it is a plant virus (i.e., not a human pathogen) and in even higher abundance than human adenovirus [10]. 

The high tolerance of PMMoV to the wastewater treatment process, as well as its significant abundance and concentration, initiated our hypothesis of proposing it as a potential indicator of human enteric viruses, of particular interest the highly persistent human adenovirus causing gastroenteritis. Accordingly, monitoring the possible correlation between PMMoV and human adenovirus is of critical importance. Therefore, the present study was performed for the quantification of PMMoV and HAdV in the influents of 3 wastewater treatment plants in Riyadh, Saudi Arabia, over a one-year period. Furthermore, PMMoV quantification was used for the assessment of its probable correlation to the highly abundant and persistent enteric virus, HAdV, in sewage water. The impact of environmental conditions on the concentration of both PMMoV and HAdV was also investigated.

## 2. Materials and Methods

### 2.1. Samples Collection

From January to December 2022, 36 untreated wastewater samples were collected monthly at a rate of three samples per month from three different wastewater treatment plants (WWTPs) in Riyadh, Saudi Arabia. The sampling locations were King Saud University wastewater treatment plants (KSU-WWTP), Manfoha wastewater treatment plants (MN-WWTP), and Embassy Quarter wastewater treatment plants (EMB-WWTP) in Riyadh, Saudi Arabia. In sterile 200 mL plastic bottles (wastewater sampling was conducted in triplicates, i.e., 3 × 200 mL per each sampling point), samples were collected and transferred to the laboratory on dry ice. The data for temperature, relative humidity, and wind speed were collected on each sample day from the accuweather website (https://www.accuweather.com/en/sa/riyadh, last accessed on 31 December 2022) in order to study the influence of the weather on viral persistence.

### 2.2. Viral Concentration

Pepper mild mottle virus (PMMoV) and adenovirus (Adv) were concentrated using the polyethylene glycol (PEG) precipitation process [23]. Briefly, 200 mL of treated wastewater was combined with 25 mL of glycine buffer (0.05 M glycine and 0.3 g/L beef extract) to detach virions bound to the organic material, and the pH was adjusted to 9.6 with 1 M NaOH before centrifugation at 8000× *g* for 30 min. Afterwards, the supernatant was withdrawn using sterile syringe then filtered using Millipore filter (0.22 μm) for the removal of bacterial cells and the other unfavorable debris. The filtrate was then treated with PEG 8000 (80 g/L) and sodium chloride (17.5 g/L) followed by stirring over magnetic stirrer overnight (100 rpm) at room temperature for viral precipitation. Centrifugation of the filtrate was then performed at 13,000× *g* for harvesting viral particles, and then, the pellet was eluted in 1 mL phosphate buffer saline (PBS) and finally stored at −80 °C for further experimentations.

### 2.3. Viral Nucleic Acid Extraction

HAdV DNA was extracted using DNeasy PowerWater Kit (Qiagen GmbH, Hilden, Germany) following the manufacturer’s instructions. PMMoV RNA was extracted using ZymoBIOMICS RNA Miniprep kit (ZymoBIOMICS™, Zymo Research, Irvine, CA, USA) [24]. PMMoV RNA was reverse transcribed using Sensiscript Reverse Transcription (RT) Kit (SensRT; Qiagen GmbH, Hilden, Germany). 

### 2.4. Quantitative Polymerase Chain Reaction (qPCR) of Adenoviruses

The quantitative detection of adenoviruses was conducted using RealStar^®^ Adeno-virus PCR Kit 1.0 (altona DIAGNOSTICS, Hamburg, Germany). The kit contains the following components; Master A and Master B include all of the necessary components (PCR buffer, DNA polymerase, magnesium salt, primers, and probes) for PCR-mediated amplification and detection of HAdV based on a partial highly conserved sequence in a single reaction setup. First, the master mix of q-PCR was prepared before the reaction by mixing 5 μL of Master A with 15 µL of Master B for each reaction. In each required well of an optical 96-well reaction plate, 10 μL of the Master Mix was added, followed by 5 μL of DNA samples. The reaction conditions were as follows: one cycle at 95 °C for 10 min as a denaturation step, followed by 45 cycles at 95 °C for 15 s, and one minute at 58 °C as an amplification step. Sterile nuclease-free water was included in each set of extractions as a negative control to monitor cross-contamination, whereas the standard dilutions of HAdV provided with the kit were used to construct the standard curve to ensure the efficiency of RTqPCR efficiency. R^2^ denoted the assay performance efficiency (R^2^ = 0.987, Slope = −3.258). The detection limit of qPCR assay was determined to be <10 amplicon copies/run, which resulted from qPCR of standard serial dilutions. The virus concentration per liter (GC_L_) was calculated according to the following equation:

$${\mathrm{GC}}_{L}=\frac{\mathrm{GC}\times \mathrm{DF}}{V},$$

where GC refers to the number of genomic copies per reaction and DF denotes the dilution factor.

### 2.5. Quantitative Polymerase Chain Reaction (qPCR) of PMMoV

A DNA standard for PMMoV was prepared using the primers PM1602 5’-TGTTTCGGAAAAGGCTCTTG-3’(forward primer) and Ha-PMMV2 5’-ATTTGCTTCGGTAGGCCTCT-3’ (reverse primer), which targeted 319 bp of the genome [12]. A standard curve for the assay was developed using standard 4-fold serial dilutions in order to establish the detection limit of the assay. The detection limit of qPCR assay was determined to be 10 amplicon copies/run. Moreover, R^2^ denoted the assay performance efficiency (R^2^ = 0.999, Slope = −2.636, y-int = 34.174). RT-PCR of PMMoV was carried out using oasigTM lyophilised OneStep qRT-PCR Mastermix (Primerdesign, Eastleigh, Southampton, UK) following the manufacturer’s instructions. The reaction mixture (20 μL) contained the following components; oasigTM Lyophilised OneStep Mastermix (10 μL), forward primer (1 μL), reverse primer (1 μL), probe (1 μL), RNAse/DNAse free water (2 μL), and the template RNA (5 μL). The reaction conditions were set as follows: reverse transcription (RT) at 42 °C for 10 min, RT enzyme inactivation at 95 °C for 2 min, 50 cycles programmed as 95 °C for 15 s as denaturation step, 50 °C for 30 s as annealing step, and lastly, 72 °C for 15 s as an extension step. Nuclease-free water was also included in each run as a negative control. The primers and probe utilized in the RT-PCR reaction were listed as shown in Table 1.

### 2.6. Statistical Analysis

Pearson’s correlation coefficient matrix was used to investigate the possible correlations between the viral concentrations among various sample locations during a one year period [23]. To investigate the influence of meteorological parameters (including temperature, wind speed, and relative humidity (RH%)) on PMMoV and HAdV concentrations, a one-way analysis of variance was performed. Linear curve fitting was used to fit the relationship between distinct sample sites as dependent variables and meteorological conditions as independent variables. The XL-STAT statistics package software was used for all statistical analyses (Version 2019, Excel Add-ins soft SARL, New York, NY, USA).

## 3. Results

### 3.1. PMMoV Incidence in Raw Water of WWTPs

**Figure 1:**
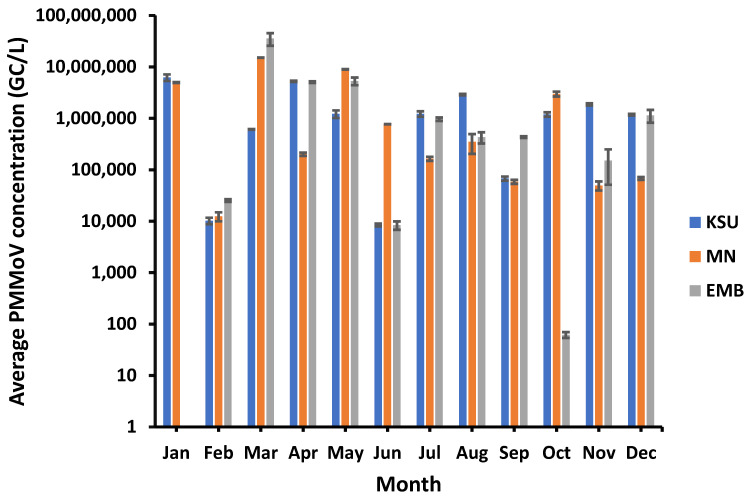
PMMoV concentration in raw water of the different WWTPs.

Overall, PMMoV was found detected in 94.4% of raw water samples with the highest PMMoV prevalence at MN-WWTP (100%), followed by KSU-WWTP and EMB-WWTP (91.6%). However, PMMoV showed a large variation in virus concentration, ranging from 62 copy/liter (GC/L), occurring in October, to 3.5 × 10^7^ GC/L, detected in March, in the raw water of EMB-WWTP. However, the highest PMMoV concentration detected in KSU-WWTP was 6.22 × 10^6^ GC/L (in January) and the lowest concentration of PMMoV was 8.47 × 10^3^ GC/L (in June). Compared to PMMoV concentration in KSU-WWTP raw water, PMMoV was found in a higher concentration in MN-WWTP ranging from 1.24 × 10^4^ (in February)–1.51 × 10^7^ GC/L (in March)—still lower than that in EMB-WWTP raw water, however (Figure 1). On the contrary, PMMoV was only undetected in January in the EMB-WWTP. 

### 3.2. Seasonal Impact on PMMoV Concentration

**Figure 2:**
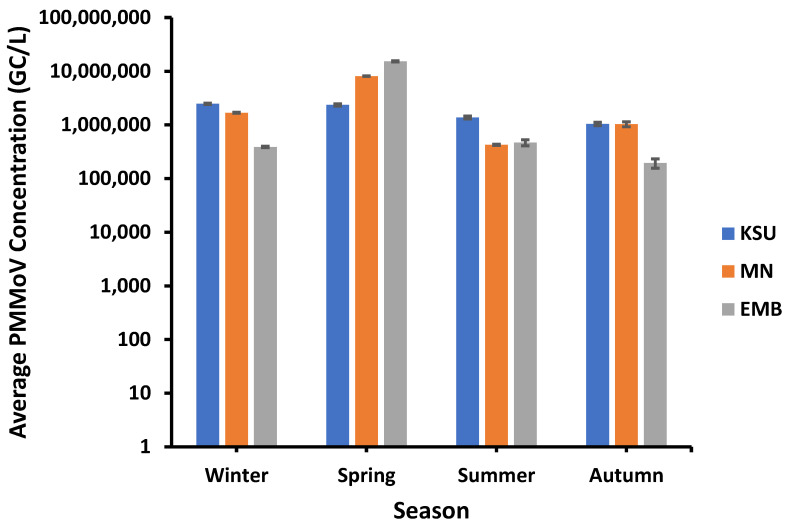
Seasonal influence on PMMoV concentration.

PMMoV showed higher concentrations in spring than the other seasons. For instance, the highest PMMoV average concentrations of approx. 8.09 × 10^6^ GC/L and 1.53 × 10^7^ GC/L were recorded in spring in both MN-WWTP and EMB-WWTP, respectively, whilst the highest average concentration of PMMoV at KSU-WWTP (ca. 2.47 × 10^6^ GC/L) was found in winter (Figure 2). 

On the other hand, the lowest PMMoV average concentrations of 1.94 × 10^5^ GC/L and 1.04 × 10^6^ was observed in autumn in EMP-WWTP and KSU-WWTP, respectively. Although it was unlikely, the lowest average PMMoV concentration in MN-WWTP (4.27 × 10^5^ GC/L) was detected in summer. Moreover, the inter-WWTPs correlation was tested in terms of seasonal PMMoV average concentration. Remarkably, the PMMoV concentration variation among seasons in EMB-WWTP was of a higher association with that occurring in MN-WWTP (r = 0.9889) than that occurring between KSU-WWTP and MN-WWTP (r = 0.598) and between KSU-WWTP and EMB-WWTP (r = 0.546). 

### 3.3. Temparature Variation Impact on PMMoV Concentration

**Figure 3:**
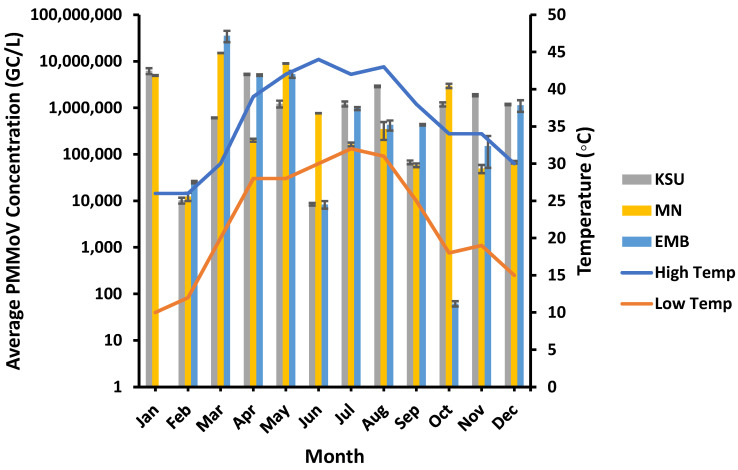
Temperature variation influence on the PMMoV concentration in wastewater samples. “Low Temp” referred to the average low temperature and “High Temp” refers to the average high temperature.

The highest PMMoV concentrations (6.22 × 10^6^, 1.51 × 10^7^, and 3.55 × 10^7^ GC/L) generally occurred in mid-winter and early spring at low temperatures (high: 26–30 °C, low: 10–20 °C), particularly in January and March at KSU-WWTP, MN-WWTP, and EMB-WWTP, respectively (Figure 3). Conversely, the lowest PMMoV concentrations were detected at higher temperatures (high: 34 °C and 44 °C, low: 18 °C and 30 °C) at EMB-WWTP and KSU-WWTP, respectively. Although it was unlikely, the lowest PMMoV concentration at MN-WWTP was found at lower temperatures (high: 26 °C, low: 12 °C). Despite the observed pattern, the high and low temperature ranges were found to have an insignificant influence on the prevalence of PMMoV (*p* > 0.05) at EMB-, MN-, and KSU-WWTPs (T_H_: R^2^ = 0.04, 0.043, and 0.02, respectively, and T_L_: R^2^ = 0.0005, 0.014, and 0.021, respectively; Table 2).

### 3.4. Humidity Impact on PMMoV Concentration

**Figure 4:**
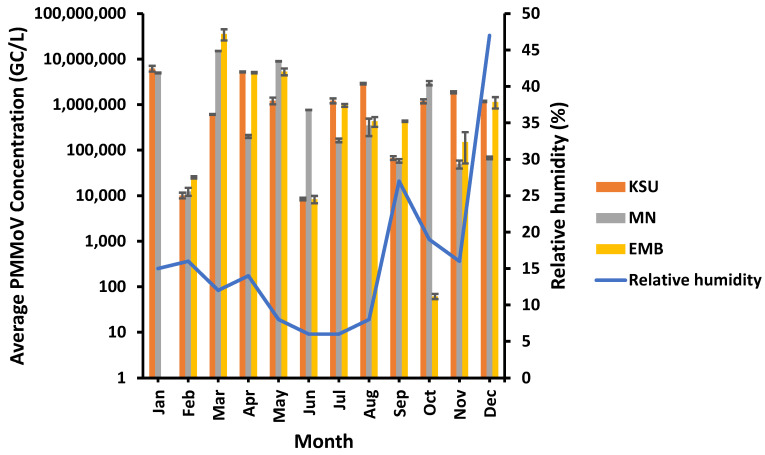
Relative humidity impact on the PMMoV concentration in raw water samples.

PMMoV was observed of the highest concentrations at a moderate relative humidity of 12% at MN- and EMB-WWTPs and 15% at KSU-WWTP in March and January, respectively (Figure 4). On the contrary, the lowest PMMoV concentrations were detected at a higher relative humidity of 16% and 19% at EMB-WWTP and MN-WWTP, respectively. Although it was unlikely, the lowest PMMoV concentration at KSU-WWTP was found at the lowest relative humidity of 6%. Consequently, PMMoV concentrations were found to be insignificantly affected by relative humidity (*p* > 0.05) at EMB-, MN-, and KSU-WWTPs (R^2^ = 0.019, 0.0577, and 0.0113, respectively; Table 2).

### 3.5. Wind Speed Influenced PMMoV Incidence

**Figure 5:**
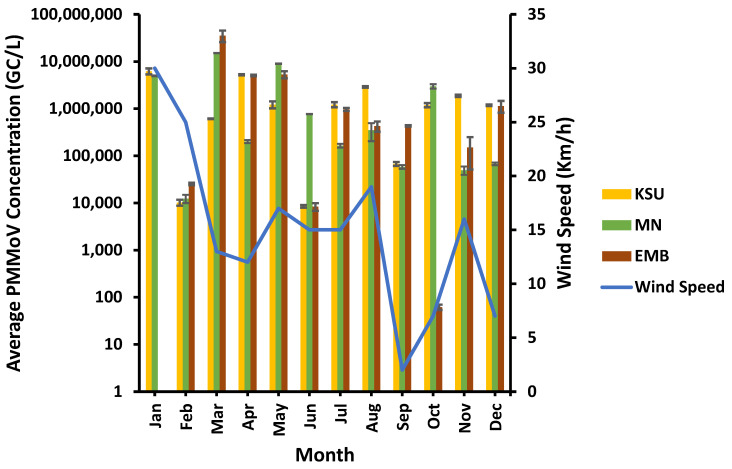
Influence of wind speed variation on the PMMoV concentration.

PMMoV was found of the greatest concentrations at a moderate wind speed of 13 Km/h at MN- and EMB-WWTPs and at a high wind speed of 30 Km/h at KSU-WWTP in March and January, respectively (Figure 5), whereas the lowest PMMoV concentrations were detected at different wind speed levels varying from a low wind speed of 7 Km/h at EMB-WWTP to a moderate wind speed of 15 Km/h at KSU-WWTP to a high wind speed level of 25 Km/h at MN-WWTP. Therefore, wind speed showed no significant influence on PMMoV concentration (*p* > 0.05) at EMB-, MN-, and KSU-WWTPs (R^2^ = 0.0087, 0.0117, and 0.1976, respectively; Table 2).

### 3.6. HAdV Incidence in Raw Water of WWTPs

**Figure 6:**
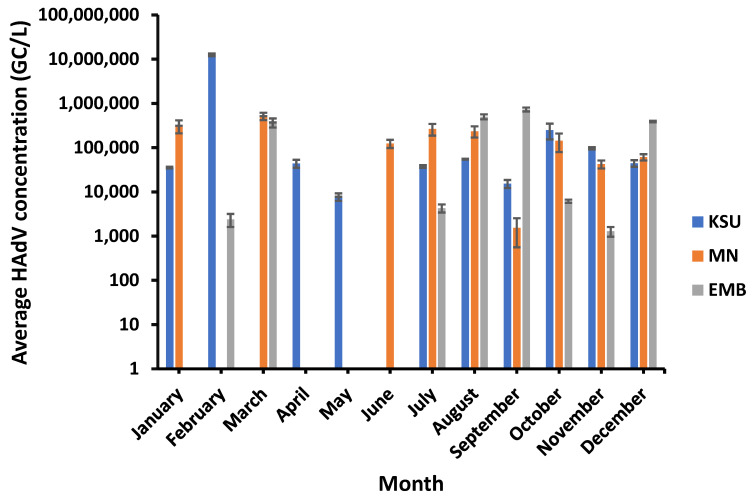
HAdV concentration in raw water of the different WWTPs.

HAdVs were found positive in 75% of the samples, with the highest prevalence (83%) at KSU-WWTP and lowest prevalence (66.6%) at EMB-WWTP. Overall, HAdV ranged from 1.29 × 10^3^ GC/L, which occurred in November, to 1.26 × 10^7^ GC/L, recorded in February, in the raw water of EMB-WWTP and KSU-WWTP, respectively. However, the highest HAdV concentration detected in MN-WWTP was 5.15 × 10^5^ GC/L (in March), and the lowest concentration of HAdV was 1.55 × 10^3^ GC/L (in September). Compared to PMMoV concentration in MN-WWTP raw water, HAdV was found in a higher concentration in EMB-WWTP of 7.32 × 10^5^ GC/L (in September) but still lower than that in KSU-WWTP raw water (Figure 6). On the other hand, HAdV was undetected in KSU-WWTP (in March and June), in MN-WWTP (in February, April, and May), and in EMB-WWTP (in January, April, May, and June). 

### 3.7. Seasonal Impact on HAdV Concentration

**Figure 7:**
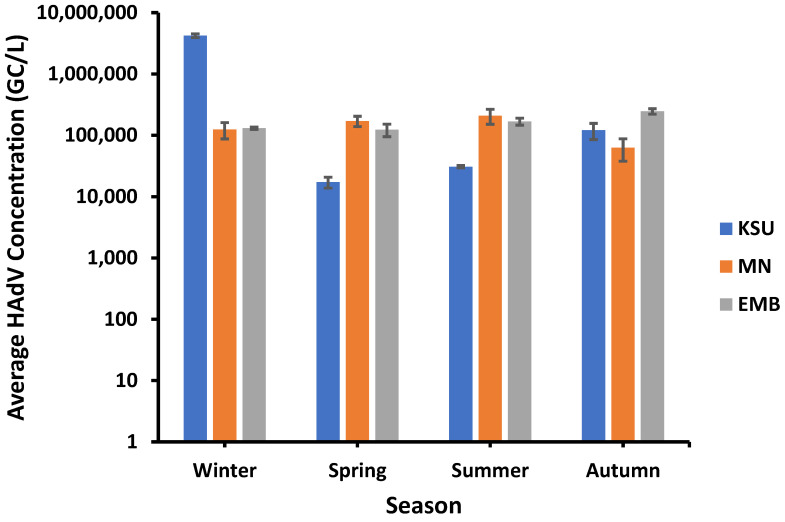
Seasonal influence on HAdV concentration 3.8. Temperature variation impact on HAdV concentration.

HAdV displayed the lowest concentrations in spring rather than the other seasons. For instance, the lowest HAdV average concentrations of approx. 1.73 × 10^4^ GC/L and 1.24 × 10^5^ GC/L were recorded in spring in both KSU-WWTP and EMB-WWTP, respectively, whilst the lowest average concentration of HAdV at MN-WWTP (ca. 6.27 × 10^4^ GC/L) was detected in Autumn (Figure 7). 

On the other hand, the highest HAdV average concentrations were recorded in all seasons except for spring; however, it varied according to the raw water source. The highest average HAdV concentration was detected in KSU-WWTP (about 4.23 × 10^6^ GC/L) in winter, followed by EMB-WWTP (2.46 × 10^5^ GC/L) in autumn and MN-WWTP (2.08 × 10^5^ GC/L) in summer. Moreover, the inter-WWTPs correlation was tested in terms of seasonal HAdV average concentration. The HAdV concentration variation among seasons in EMB-WWTP was of a higher negative association (i.e., inverse correlation) with that occurring in MN-WWTP (*r* = −0.6436) than that occurring between KSU-WWTP and EMB-WWTP (*r* = −0.4158) and between KSU-WWTP and MN-WWTP (*r* = −0.2046). 

**Figure 8:**
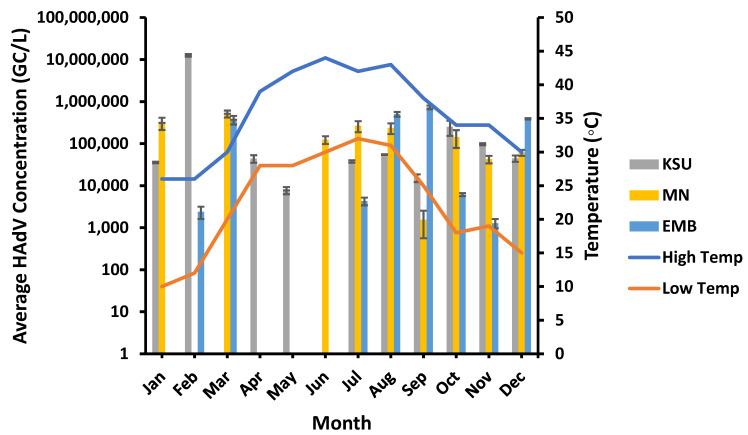
Temperature variation influence on the HAdV concentration in wastewater samples. “Low Temp” refers to the average low temperature, and “High Temp” refers to the average high temperature.

The highest HadV concentrations (1.26 × 10^7^, 5.15 × 10^5^ and 7.32 × 10^5^ GC/L) occurred in late winter, early spring, and early autumn at low and moderate temperatures (high: 26 °C, 30 °C, and 38 °C, low: 12 °C, 20 °C, and 25 °C), particularly in February, March, and September at KSU-WWTP, MN-WWTP, and EMB-WWTP, respectively (Figure 8). The lowest HAdV concentrations were detected at higher temperatures (high: 42 °C and 38 °C, low: 28 °C and 25 °C) at KSU-WWTP and MN-WWTP, respectively. Although it was unlikely, the lowest HAdV concentration at EMB-WWTP was found at a lower temperature (high: 34 °C, low: 19 °C). Despite the observed pattern, the high and low temperature ranges showed no significant impact on the concentration of HAdV (*p* > 0.05) at EMB-, MN-, and KSU-WWTPs (T_H_: R^2^ = 0.0032, 0.033, and 0.217, respectively, and T_L_: R^2^ = 0.0137, 0.003, and 0.184, respectively; Table 3).

### 3.8. Humidity Impact on HAdV Prevalence

**Figure 9:**
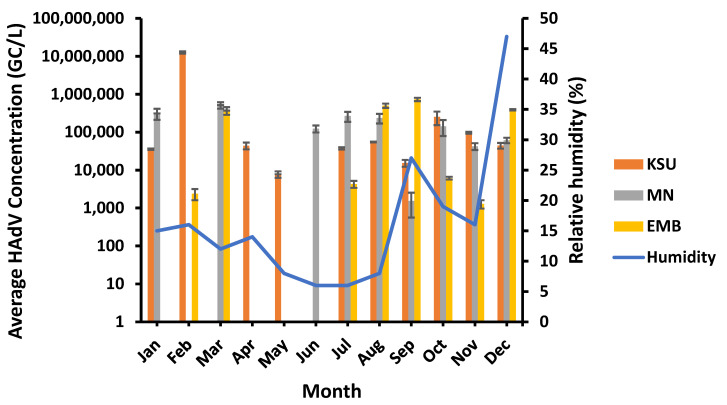
Relative humidity influence on the HAdV concentration.

HAdV was observed in the highest concentrations at a moderate relative humidity of 12% and 16% at MN- and KSU-WWTPs (in March and February, respectively) and at a higher relative humidity of 27% in September at EMB-WWTP (Figure 9). The lowest HAdV concentrations were detected at various relative humidity levels ranging from low (8%) at KSU-WWTP to moderate (16%) at EMB-WWTP to high (27%) at MN-WWTP. Therefore, HAdV concentrations were not significantly influenced by the relative humidity (*p* > 0.05) at EMB-, MN-, and KSU-WWTPs (R^2^ = 0.19, 0.089, and 3.85 × 10^−6^, respectively; Table 3).

### 3.9. Wind Speed Impact on HAdV Concentration

**Figure 10:**
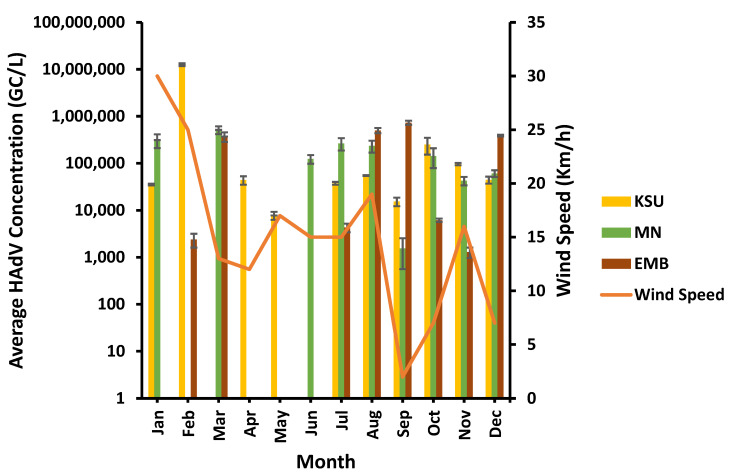
Influence of wind speed on HAdV concentration.

HAdV was found in the greatest concentrations at various wind speed levels. HAdV’s highest concentration was observed at low (2 Km/h), moderate (13 Km/h), and high (25 Km/h) wind speed levels at EMB-, MN-, and KSU-WWTP, respectively (Figure 10). The lowest HAdV concentrations were detected at a low wind speed of 2 Km/h in MN-WWTP and a moderate wind speed of 16–17 Km/h at EMB- and KSU-WWTP. Consequently, wind speed displayed no significant impact on HAdV concentrations (*p* > 0.05) at EMB-, MN-, and KSU-WWTPs (R^2^ = 0.25186, 0.056, and 0.25186, respectively; Table 3).

### 3.10. Correlation of the Surrogate PMMoV Concentration to HAdV Concentration

The insignificant impact of the entirety of meteorological factors on both PMMoV and HAdV concentrations led us to examine the correlation between PMMoV and HAdV concentrations at the different sampling locations. However, a concentration variation of PMMoV showed moderate positive correlation to that of HAdV only at MN-WWTP (r = 0.6148), whereas weak positive and negative correlations between PMMoV and HAdV concentrations were detected at EMB- and KSU-WWTPs, respectively (r = 0.207 and −0.28, respectively). 

On the other hand, the seasonal impact on PMMoV-to-HAdV concentrations’ correlation was examined. The seasonal factor showed a strong positive correlation (r = 0.918) of the viral surrogate (PMMoV) to the pathogenic human virus (HAdV) at KSU-WWTP and a moderate positive correlation (r = 0.6401) at EMB-WWTP, whereas it showed a negative moderate correlation at MN-WWTP (r = −0.5069). 

## 4. Discussion

Public exposure to wastewater is frequently occurring by different means, including agriculture, recreational activities, etc., and is usually associated with a high infection risk [25]. Therefore, wastewater surveillance is essential to combat the probable outbreaks due to enteric virus prevalence and infectivity and promote vaccination initiatives. For instance, wastewater monitoring was instrumental in poliovirus control in UK, Finland, the Netherlands, etc. [26,27]. Lately, poliovirus was recorded in Jerusalem sewage water in 2022 that initiated vaccination campaigns for non-fully immunized individuals, resulting in almost no paralysis cases with a single paralyzed unvaccinated child [26,28]. However, surveillance studies are also looking for safe viral indicators for enteric virus-mediated fecal pollution that is of high abundance, concentration, and well-correlated with that of the enteric viruses. PMMoV is more abundant in wastewater than viruses that cause human disease, most likely because PMMoV in human feces is of dietary origin (from peppers and their processed products, such as hot sauce and curry) and is excreted from large, healthy human populations [29]. 

This potentially high prevalence of PMMoV is required for a successful viral surrogate acting as a fecal pollution indicator [30]. However, a significantly high concentration of this viral indicator is also a must. A study was conducted in Japan during the period between Dec 2015 to Jan 2017 and found that PMMoV concentrations were about 10^5.35 ± 0.48^ copies/L for raw water samples [31]. Moreover, PMMoV recorded the highest concentration (annual mean concentration of 3.7–4.4 × 10^6^ copies/L; range: 6.22 × 10^6^ to 3.5 × 10^7^ GC/L) among eleven different viruses at two WWTPs in southern Arizona over a 12 month period, from August 2011 to July 2012 [29]. In the same manner, high concentrations were detected in the entirety of the WWTPs raw water investigated in the current study (6.22 × 10^6^–3.5 × 10^7^ copies/L), which even agrees with other studies conducted in New Zealand (7.1 ± 0.5 log10 GC/L) [32], Vietnam (5.5 × 10^6^–7.2 × 10^6^ copies/L) [33], and Italy (1.2 × 107 GC/L) [34]. However, a significantly low PMMoV concentration (62 copies/L) was detected once at EMB-WWTP throughout the study. This could be due to a probable meteorological impact or poor viral concentration and/or recovery. An efficient viral concentration method is a mandate for potential recovery of the waterborne viruses, characterized with potentially low concentrations beyond the molecular detectability [24]. For instance, polyethylene glycol (PEG) precipitation was found with higher efficiency at recovering non-enveloped viruses than enveloped viruses from the aquatic environment [35]. Furthermore, a PEG-mediated secondary concentration even yielded the highest poliovirus recovery [36]. In the same manner, our study involved PEG as the virus concentration method for achieving better viral recovery. 

The potentially high concentration of PMMoV depicted major characteristics required for a viral surrogate; however, comparing it to a potential prevalent enteric virus is also demanded. In such regard, several studies reported the highest persistence of HAdV, a potential human enteric virus, among other enteric viruses in almost all water sources, including the raw water [23,30]. Our study demonstrated high viral concentration ranging from 1.29 × 10^3^ GC/L to 1.26 × 10^7^ GC/L, which agrees with a previous annual study conducted in Egypt showing comparable HAdV concentrations in influent samples (1.5 × 10^4^–1.5 × 10^7^ GC/L) [34]. Moreover, HAdV recorded a relative concentration of 2.37 × 10^5^ genome/L in the Eastern Cape, South Africa, which is close to the average HAdV concentration in our study. However, this indicates even higher HAdV prevalence in WWTPs of Eastern Cape, South Africa since it was an HAdV concentration in effluent samples—indicating HadV persistence [37].

Therefore, the current study investigated if such HadV persistence was influenced by seasonal variations. The impact of seasonality and associated meteorological factors were found entirely insignificant on the detected HadV concentrations in all locations. In the same manner, no seasonal trend was recorded for adenovirus concentration in wastewater samples [37]. Moreover, high concentrations of HadV were reported in the majority of samples, with no observed seasonal patterns [38]. HadV displayed high concentrations with insignificant seasonal influences (required for potential enteric virus persistence), which support our hypothesis for comparison with our viral surrogate, PMMoV. Therefore, PMMoV was also investigated to determine if its concentration was influenced by seasonal differences. Our study found an insignificant influence of seasonality and associated meteorological factors on PMMoV concentrations in all locations. In the same manner, PMMoV concentration depicted no clear seasonal variation among the wastewater samples collected in Kentucky, USA [39], as well as in Okinawa, Japan [40]. Moreover, a study conducted in Mexico observed no significant correlation between PMMoV occurrence by season or water type [14], which agrees with our findings.

The insignificant impact of meteorological factors on PMMoV concentration, as well as the lack of seasonal PMMoV distribution pattern, supports it being a potential viral surrogate. However, the PMMoV concentration correlation to that of the human enteric virus HAdV is a must for a successful fecal indicator. The concentration variation of PMMoV showed a positive correlation to that of HAdV at MN- and EMB-WWTPs. Likewise, there was a significant positive correlation between the concentrations of human enteric viruses and PMMoV, proposing PMMoV as a potential indicator of the presence of enteric viruses in the Bagmati River water [41]. Moreover, it was reported that minimum PMMoV concentrations were similar to the minimum concentrations of AdV, HPyV, and NoV, whereas the PMMoV maximum and median concentrations were at least one-fold greater than these human viruses, supporting the PMMoV-to-enteric viruses’ correlation [16].

## 5. Conclusions

The characteristics of a successful viral surrogate for application in fecal pollution monitoring, including the high concentration, lack of seasonality, and lack of meteorological factors’ impact on virus concentration and eventual correlation with highly persistent human enteric viral pathogens, were all found in the case of PMMoV. Consequently, PMMoV could be considered a successful fecal pollution indicator for enteric viruses in wastewater. The present study underwent a limitation of using a single enteric virus (HAdV) for comparison with the viral surrogate (PMMoV). However, monitoring of other persistent enteric viruses and comparison with PMMoV is recommended for a more effective application of PMMoV as a viral surrogate indicator. Another limitation was the single water type (i.e., WWTPs’ influents) used in the current study. The inclusion of other water resources, including recreational water, surface water, and treated water, will further broaden this viral surrogate application in monitoring possible fecal contamination. Our study was a single-year study; therefore, long-term studies are highly recommended. Such studies are essential to determine the putative impact of meteorological factors on viruses’ concentrations in the aquatic environments that could also impact the reliability of our proposed fecal pollution indicator, PMMoV, and uncover any possible fluctuations due to meteorological conditions’ variation over years.

## Figures and Tables

**Table 1:** Primers and probes used in the RT-PCR reaction.

Function	Primer/Probe	Sequence (5′–3′)	Reference
Forward primer	PMMV-FP1-rev	GAGTGGTTTGACCTTAACGTTTGA	[13]
Reverse primer	PMMV-RP1	TTGTCGGTTGCAATGCAAGT	
TaqMan probe	PMMV-Probe1	FAM-CCTACCGAAGCAAATG-BHQ1	

**Table 2:** Significance of environmental factors’ influences on the concentration of PMMoV.

Environmental Factor	Location	*R* ^2^	RMSE	Equation
High temperature (T_H_)	EMB	0.04	10,348,141.3	*GC*_MMoV_ = 15,109,644.77 − 309,151.929∗T_H_
	MN	0.043	4,873,605.45	*GC*_MMoV_ = 8,161,620.14 − 150,118.32∗T_H_
	KSU	0.02	2,093,242.14	*GC*_MMoV_ = 3,392,512.85 − 44,380.8∗T_H_
Low temperature (T_L_)	EMB	0.0005	10,563,315.4	*GC*_MMoV_ = 4,741,219.53 − 29,462.4∗T_L_
	MN	0.014	4,947,281.5	*GC*_MMoV_ = 4,451,911.54 − 73,634.845∗T_L_
	KSU	0.021	2,093,227.55	*GC*_MMoV_ = 2,660,847.35 −38,115.66∗T_L_
Relative humidity (RH%)	EMB	0.019	10,463,904.3	*GC*_MMoV_ = 6,057,248.5 − 122,104.49∗RH%
	MN	0.0577	4,836,788.65	*GC*_MMoV_ = 4,419,586.49 − 99,722.88∗RH%
	KSU	0.0113	2,103,541.09	*GC*_MMoV_ = 2,112,875.73 − 18,759.48∗RH%
Wind speed (WS)	EMB	0.0087	10,519,736.4	*GC*_MMoV_ = 5,891,981.11 − 121,938.55∗WS
	MN	0.0117	4,953,237.27	*GC*_MMoV_ = 1,818,076.995 + 66,695.93∗WS
	KSU	0.1976	1,895,054.97	*GC*_MMoV_ = 86,931.8 + 116,134.766∗T_H_

**Table 3:** Significance of environmental factors’ influences on the concentration of HAdV.

Environmental Factor	Location	*R* ^2^	RMSE	Equation
High temperature (T_H_)	EMB	0.0032	271,615.12	*C*_HAdV_ = 87,417.63 + 2238.59∗T_H_
	MN	0.033	167,124.71	*C*_HAdV_ = 302,316.98 − 4501.29∗T_H_
	KSU	0.217	3,362,628.5	*C*_HAdV_ = 10,235,239.19 − 256,157.73∗T_H_
Low temperature (T_L_)	EMB	0.0137	270,183.17	*C*_HAdV_ = 78,793.66 − 3961.216∗T_L_
	MN	0.003	169,765.37	*C*_HRV_ = 166,012.86 − 1085.46∗T_L_
	KSU	0.184	3,431,201.9	*C*_HAdV_ = 5,630,067.79 − 202,886.02∗T_L_
Relative humidity (RH%)	EMB	0.19	244,722.61	*C*_HAdV_ = 7129.66 + 9905.02∗RH%
	MN	0.089	162,234.35	*C*_HAdV_ = 210,162.59 − 4230.42∗RH%
	KSU	3.87 × 10^−6^	3,799,335.2	*C*_HAdV_ = 1,109,019.18 − 623.04∗RH%
Wind speed (WS)	EMB	0.25186	235,313.61	*C*_HAdV_ = 417,372.29 − 16,861.446∗WS
	MN	0.056	165,155.66	*C*_HAdV_ = 1.36 − 5.27× 10^−2^∗WS
	KSU	0.25186	235,313.61	*C*_HAdV_ = 68,037.21 + 4970.8∗WS

*C* denotes the concentration of virus. RMSE denotes the root mean squared error.

## Data Availability

The data presented in this study are available in the article.
